# Bone turnover decreases and bone structure improves during treatment with weekly high-dose methylprednisolone for 12 weeks in Graves’ orbitopathy

**DOI:** 10.1007/s12020-023-03494-5

**Published:** 2023-09-07

**Authors:** Torben Harsløf, Rawan Hikmet, Eva Ebbehøj, Bente Langdahl

**Affiliations:** 1 Department of Endocrinology and Internal Medicine, Aarhus University Hospital, Aarhus, Denmark; 2 Department of Clinical Medicine, Aarhus University Hospital, Aarhus, Denmark

**Keywords:** Graves’ orbitopathy, Glucocorticoid, Bone mineral density, Bone structure

## Abstract

**Purpose:**

Weekly treatment with the intravenous glucocorticoid methylprednisolone for 12 weeks is mainstay in the treatment of Graves’ orbitopathy but may decrease bone mass and impair bone structure. We therefore investigated bone turnover, -mass and -structure during the treatment cause in these patients.

**Methods:**

We included 32 patients with Graves’ orbitopathy scheduled for treatment with methylprednisolone. Bone turnover and thyroid function was measured at baseline and after 3, 9, 12, and 24 weeks, bone mineral density (BMD) was measured using dual x-ray absorptiometry at baseline and after 12 and 24 weeks, and bone structure was measured using high-resolution peripheral quantitative computed tomography at baseline and after 12 weeks.

**Results:**

Bone turnover and tri-iodothyronine decreased throughout the study. Cortical volumetric BMD at both the radius and tibia increased significantly by 0.98 ± 0.38% (*p* = 0.01) and 1.35 ± 0.50% (*p* = 0.01), respectively and cortical porosity at both the radius and tibia decreased significantly by −7.67 ± 3.13% (*p* = 0.04) and −3.30 ± 2.17% (*p* = 0.04), respectively. Bone mineral density was stable during the first 12 weeks but increased significantly by 2.26 ± 3.61% at the femoral neck (*p* < 0.01) and by 2.24 ± 4.24% at the total hip towards week 24 (*p* = 0.02). Stratified analyses suggested that remission of hyperthyroidism was the most important determinant of changes in bone turnover, bone mass and structure.

**Conclusion:**

During a 12-week course of high-dose intravenous methylprednisolone bone turnover and cortical porosity decreased and during 24 weeks follow up bone mineral density increased. In terms of bone, methylprednisolone therefore is a safe treatment for Graves’ orbitopathy.

## Introduction

Graves’ orbitopathy (GO) is an autoimmune condition associated with autoimmune hyperthyroidism (Graves’ disease). Mainstay in the treatment of GO is intravenous glucocorticoid (GC). A treatment regimen with methylprednisolone (MP) once weekly for 12 weeks (500 mg weekly for 6 weeks followed by 250 mg weekly for 6 weeks) summing up to a total dose of 4500 mg is proven to be effective [1] in severe GO and is therefore prescribed for most patients in which treatment is indicated, however, other regimens using higher or lower doses are sometimes used. Long-term treatment with GC may cause side effects [2]. One side effect is a decrease in bone mineral density (BMD) owing to an increased bone resorption and a decreased bone formation [3, 4] which in turn increases risk of fractures [5] –in particular vertebral fractures [6]. A daily intake of GC of more than 5 mg prednisolone for at least three months increases fracture risk and decreases BMD [7]. Furthermore, treatment with GCs affects bone structure [8] which may increase fracture risk independently of BMD. There are studies, however, showing that the decrease in BMD with GC treatment for up to 20 weeks is largely reversible [9] and that fracture risk is more closely related to daily- than to cumulative dose [10]. Accordingly, it has been shown, that intermittent high dose GC as an adjuvant to chemotherapy in breast cancer does not decrease BMD [11]. Finally treatment of hyperthyroidism increases BMD [12]. Therefore, in patients with Graves’ disease treated with MP for GO there are factors that could affect bone either way. In order to fully elucidate changes in bone turnover, -mass, and –quality during the treatment course we therefore conducted a clinical trial to investigate the effect of high dose intravenous MP treatment for GO for 12 weeks on these parameters. As potential detrimental effects of MP on bone could be reversible follow up was an additional 12 weeks, making the total study duration 24 weeks.

## Methods

### Study design

A prospective, clinical cohort study.

### Participants

We included 32 adults above the age of 18 years (no upper limit) diagnosed with GO. All patients had been examined at the Department of Ophthalmology, Aarhus University Hospital, Denmark and were diagnosed with active GO with a clinical activity score ≥ 3 [13]. Thereby they fulfilled the criteria for intravenous MP according to national guidelines. Inclusion criterium was planned treatment with intravenous MP 500 mg once weekly for 6 weeks and 250 mg once weekly for 6 weeks. We excluded patients who were currently receiving osteoporosis treatment, or had received oral GCs within three months prior to inclusion but we did not exclude patients based on whether they took supplementation with calcium and/or vitamin D. We furthermore excluded patients with primary hyperparathyroidism, hypoparathyroidism, liver disease, chronic kidney disease with eGFR <30 mL/min, or vitamin D < 20 mmol/L. Moreover, we excluded patients during the study if standard treatment protocol for GO was altered or extended due to insufficient treatment response.

### Procedures

We recruited participants from the Department of Endocrinology and Internal Medicine at Aarhus University Hospital, Denmark. We informed potential participants about the study during a visit at the outpatient clinic. The trial was conducted in accordance with Note for Guidance on Good Clinical Practice (CPMP/ICH/135/95), was approved by the Danish Data Protection Agency and the Regional Ethics Committee, and was registered with clinicaltrials.gov (NCT03122847) prior to recruitment of participants. All participants gave informed consent prior to study procedures. Treatment with methylprednisolone was initiated at baseline. Upon referral to treatment and before baseline (up to 1 week) we collected blood samples measuring thyroid stimulating hormone (TSH), tri-iodothyronine (T3), thyroxine (T4), thyrotropin receptor antibody (TRAb) creatinine, sodium, potassium, parathyroid hormone (PTH), ionised-calcium, vitamin D, liver transaminase, alkaline phosphatase and bilirubin to confirm thyroid disease and to rule out presence of exclusion criteria. We also measured the bone turnover markers (BTM) C-terminal telopeptide type 1 collagen (CTx) and N-terminal procollagen type 1 propeptide (P1NP). Participants were given an infusion with MP 500 mg weekly for six weeks and afterwards 250 mg weekly for six weeks. This treatment is in accordance with national guidelines for GO. Furthermore, participants were given 800 mg calcium and 38 µg 25-OH-vitamin D3 daily but proton pump inhibitors were not routinely prescribed. Thyrotoxicosis was treated using either balanced therapy with either thiamazole or propylthiouracil or block-replacement therapy at the discretion of the treating physicians.

We collected BTMs at week 3, week 9, week 12 and week 24 for batch analyses as well as TSH, T3, T4, and TRAb at baseline, week 3, week 12 and week 24 (TRAB was not measured at week 3). We analysed CTx and P1NP using an electrochemiluminiscence immuno assay on a COBAS 8000 (Roche Diagnostics, Basel, Switzerland) with an intra-assay CV of 10% (CTx) and 8.8% (P1NP).

We also measured areal BMD at the lumbar spine (LS) L1-L4, total hip (TH), and femoral neck (FN) at baseline (no later than 10 days after initiation of treatment with MP), week 12 and week 24. Because initiation of treatment was urgent, it was not possible to do the baseline scans before treatment and therefore we performed it during the visit for the second treatment (week 1) We used the same Hologic Discovery (Hologic, Marlborough, MA, USA) scanner for all measurements. The coefficient of variation (CV) for both spine and hip BMD is approximately 1% [14]. Finally, we measured bone structure by HRpQCT (XtremeCT, Scanco Medical, Brüttisellen, Switzerland) at baseline (same time as DXA) and at week 12 (end of MP treatment). A standard carbon fibre caste was used to immobilise the arm or leg. We used a scout view to define the measurement region that started at 9.5 and 22.5 mm from the endplate of the radius and tibia, respectively. At both sites, the scan comprised 110 slices, constructing a 3D image of the bone axially with a length of 9.02 mm. We assessed the quality of each scan right away (grade 1–5) and re-scanned the subject in case of poor scan quality (grade 4–5) For quality control we performed daily phantom scans. We performed analyses of bone density and microarchitecture by using software from Scanco. In brief, we calculated trabecular bone volume per tissue volume (BV/TV) from the trabecular volume density by assuming a density of fully mineralised bone of 1.2 g hydroxyapatite (HA)/cm^3^. We measured trabecular number (Tb.N) and calculated trabecular thickness (Tb.Th) and spacing (Tb.Sp) from the BV/TV and Tb.N. We performed finite element analysis as previously described [15]. The CVs are 0.7–1.5% for density measurements, 1.0–5.5% for structural parameters, and 1.2–1.7% for finite element estimated failure load [16, 17].

### Outcomes

The primary endpoint was change in aBMD at the LS assessed by DXA. The secondary endpoints were change in aBMD at the hip, change in bone structure parameters measured by HRpQCT, change in CTx and P1NP, and change in thyroid function parameters.

### Statistics

We based the power calculation on the primary endpoint. Assuming an SD for change in LS-BMD of 3%, a level of significance of 5%, and a power of 90% we needed 30 participants to show a change of 2% in BMD using a paired samples t-test. We did not consider a change in BMD of less than 2% clinically meaningful. We calculated changes over time based on two measurements (BMD and bone structure) using paired samples t-test. Changes over time for end points with more measurements (BTMs, T3,T4 and TSH) were evaluated using a general linear model with repeated measures. These analyses were also stratified based on whether patients had normal (*n* = 11) or suppressed (*n* = 21) TSH at baseline. In case of a significant effect of time in the general linear model, we made post hoc analyses evaluating changes from baseline. In text and tables normal data are shown as mean ± SD and non-normal data as median with inter-quartile ranges. For the calculation of the median TSH level at baseline, measurements below the detection limit were set to the lowest detection limit (0.008 mIU/L).

## Results

### Baseline characteristics

**Fig. 1:**
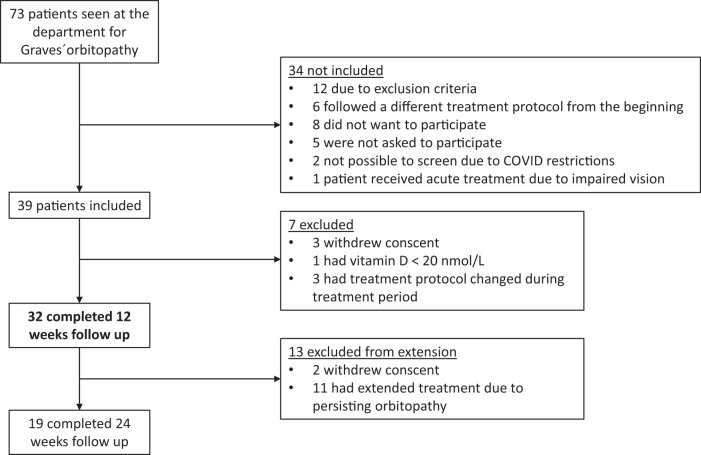
Flow of participants through the study from identification of patients at our clinic to completion of 24 weeks of follow-up. Week 12 i highlighted in boldface as the primary end point was evaluated there

In the study period, we treated 73 patients for GO at our department. Thirty-nine were included in the study, 32 completed 12 weeks of follow-up (study population) and were evaluated for the primary end-point, and 19 completed the full 24-week study period (Fig. 1)

Table 1 shows the baseline characteristics of the study population. Mean age was 52.9 ± 10.2 years, most (21) were female, and median BMI was 25.45 (IQR: 22.22–29.08) kg/m^2^. Mean levels of TSH, T3, and T4 were all within the reference ranges, however, 4 patients had overt hyperthyroidism (suppressed TSH and elevated T3 or T4) and 17 patients subclinical hyperthyroidism (suppressed TSH and normal T3 and T4). Mean vitamin D level was also well within the normal range but 9 patients had insufficient levels (data not shown). Based on the lowest T-score at either the lumbar spine, femoral neck, or total hip 3 patients had osteoporosis, 13 patients had osteopenia, and 13 patients had normal T-scores.

**Table 1:** Baseline characteristics of the study population

Study population (*N* = 32)	
Age (years)	52.9 ± 10.2
Height (cm)	169.7 ± 7.8
Weight (kg)	77.4 ± 18.3
BMI (kg/m^2^)	25.45 (22.22–29.08)
Gender	
Male	11
Female	21
Bone mineral density (g/cm^2^)	
Lumbar spine	1.00 ± 0.15
Femoral neck	0.78 ± 0.11
Total hip	0.91 ± 0.13
T-score (*N* = 29, *n* (%))	
≥−1.0	13 (44.8)
>−2.5–<−1.0	13 (44.8)
≤−2.5	3 (10.3)
Biochemistry	
CTx (µg/L)	0.70 (0.56–0.92)
P1NP (µg/L)	114 (87–157)
TSH (mIU/L)	0.04 (0.008–1.91)
T3 (nmol/L)	1.92 ± 0.67
T4 (nmol/L)	90.4 ± 35.4
TRAB (IU/L)	20 (5–31)
Vitamin D (nmol/L)	77 ± 33
Thyroid status (*n* (%))	
Euthyroid	11 (34.4)
Subclinical hyperthyroidism	17 (53.1)
Overt hyperthyroidism	4 (12.5)
Smoking status (*n* (%))	
Current	8 (25.8)
Previous	13 (41.9)
Never	10 (32.3)

Data is shown as means ± SD or medians with inter-quartile ranges

*BMI* Body mass index, *CTx* C-terminal telopeptide type 1 collagen, *P1NP* N-terminal procollagen type 1 propeptide, *TSH* Thyroid stimulating hormone, *T3* Tri-iodothyronine, *T4* Thyroxine, *TRAB* Thyroid receptor antibody

### Bone density

During the 12-week treatment period BMD changed by 1.37 ± 4.52%, −0.32 ± 4.75%, and −0.18 ± 2.62% at the LS, FN, and TH, respectively Table 2. None of these changes were statistically significant (*p* > 0.12 for all). During the entire 24-week study period, however, BMD increased significantly by 2.26 ± 3.61% at the FN (*p* < 0.01) and by 2.24 ± 4.24% at the TH (*p* = 0.02). At the LS, BMD increased non-significantly by 1.78 ± 5.44% (*p* = 0.15). In the stratified analyses, the point estimates were similar but the changes in TH- and FNBMD at 24 weeks were only statistically significant in the suppressed TSH group (data not shown). At 12 weeks 3 patients still formally had osteoporosis based on T-score, but this was only the case for one patient at 24 weeks (data not shown).

**Table 2:** Changes in aBMD during the study

	% change ± SD	*p*-value
12-week changes		
lsBMD	1.37 ± 4.52	0.13
fnBMD	−0.32 ± 4.75	0.68
thBMD	−0.18 ± 2.62	0.49
24-week changes		
lsBMD	1.78 ± 5.44	0.15
fnBMD	2.26 ± 3.61	**0.009**
thBMD	2.24 ± 4.24	**0.02**

Data is shown as mean percent change ±SD. *P*-value refers to paired sample *t*-test. Statistically significant *p*-values are highlighted in bold face

*lsBMD* Lumbar spine BMD, *fnBMD* Femoral neck BMD, *thBMD* Total hip BMD

### Bone structure

From baseline to week 12 cortical vBMD at both the radius and tibia increased significantly by 0.98 ± 0.38% (*p* = 0.01, Table 3) and 1.35 ± 0.50% (*p* = 0.01), respectively. Likewise, cortical porosity at both the radius and tibia decreased significantly by −7.67 ± 3.13% (*p* = 0.04) and −3.30 ± 2.17% (*p* = 0.04), respectively. Moreover, trabecular area at the tibia decreased significantly by 0.46 ± 1.15% (*p* = 0.04). For all other measures there were no significant changes. In the stratified analyses the pattern was the same. Cortical vBMD, -area, and –thickness increased significantly at the tibia in the TSH suppressed group as did cortical vBMD at the radius, whereas in the euthyroid group only cortical thickness at the radius increased significantly (data not shown).

**Table 3:** Change in bone structure from baseline to week 12

Radius	% change ± SD	*p*-value
Cortical area	1.25 ± 0.64	0.17
Cortical vBMD	0.98 ± 0.38	**0.01**
Cortical thickness	0.72 ± 0.49	0.53
Cortical porosity	−7.67 ± 3.13	**0.04**
Cortical pore diameter	−1.49 ± 1.36	0.19
Trabecular area	−0.29 ± 0.17	0.15
Trabecular vBMD	−0.86 ± 1.29	0.43
Trabecular BV/TV	−0.86 ± 1.29	0.48
Trabecular number	0.99 ± 2.08	0.75
Trabecular spacing	0.31 ± 2.31	0.99
Trabecular thickness	−1.17 ± 1.65	0.39
Total vBMD	0.92 ± 0.70	0.31

Tibia	% change ± SD	*p*-value
Cortical area	2.13 ± 1.09	0.08
Cortical vBMD	1.35 ± 0.50	**0.01**
Cortical thickness	1.79 ± 0.97	0.19
Cortical porosity	−3.30 ± 2.17	**0.04**
Cortical pore diameter	1.14 ± 1.44	0.46
Trabecular area	−0.46 ± 1.15	**0.04**
Trabecular vBMD	−0.47 ± 2.24	0.39
Trabecular BV/TV	−0.47 ± 2.24	0.29
Trabecular number	0.21 ± 9.13	0.85
Trabecular spacing	0.65 ± 9.01	0.62
Trabecular thickness	0.04 ± 8.35	0.87
Total vBMD	1.51 ± 3.80	0.08

Data is shown as mean percent change ±SD. *P*-value refers to paired sample *t*-test. Statistically significant *p*-values are highlighted in bold face

*vBMD* Volumetric bone mineral density, *BV/TV* Bone volume/tissue volume

### Bone turnover and thyroid function

**Fig. 2:**
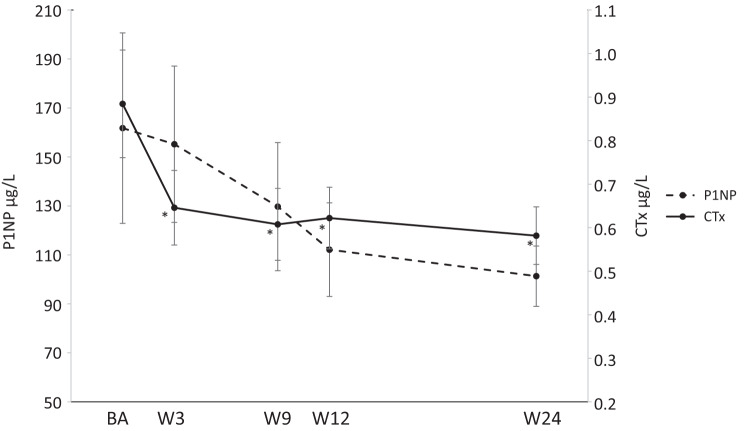
Change in levels of the bone turnover markers CTx and P1NP throughout the study period. *Statistically significant change from baseline at the 0.05 level. P1NP N-terminal procollagen type 1 propeptide, CTx C-terminal telopeptide type 1 collagen, BA Baseline, W3 Week 3, W9 Week 9, W12 Week 12, W24 Weeks 24

Using a general linear model with repeated measures CTx changed significantly over time (*p* < 0.01) (Fig. 2). Thus, at baseline CTx was 0.88 ± 0.12 µg/L (mean ± SEM) but decreased significantly towards week 3, 9, 12, and 24 to 0.65 ± 0.09 µg/L, 0.61 ± 0.08 µg/L, 0.62 ± 0.07 µg/L, and 0.58 ± 0.07 µg/L, respectively (*p* < 0.01 for all). P1NP on the other hand, did only change borderline significantly over time (*p* = 0.06) with decreases from baseline to week 9, 12, and 24. In the stratified analyses CTx decreased significantly over time in the TSH suppressed group with significant changes from baseline towards all later measurements. In the euthyroid group, however, CTx did not change significantly. Regarding P1NP the pattern was the same although it did not reach statistical significance (data not shown).

**Fig. 3:**
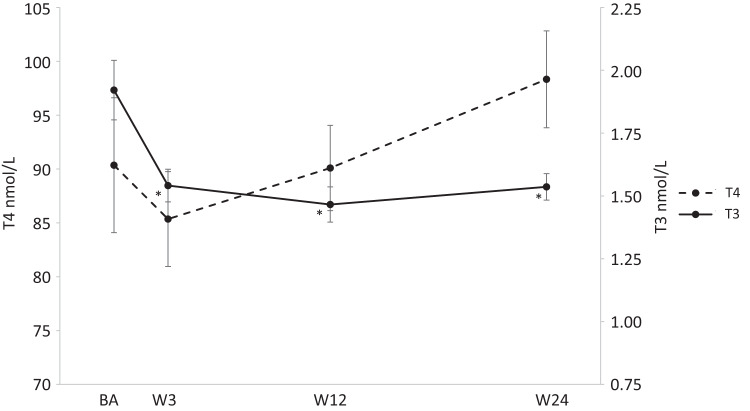
Change in levels of T3 and T4 throughout the study period. *Statistically significant change from baseline at the 0.05 level. T3 Tri-iodothyronine, T4 Thyroxine, BA Baseline, W3 Week 3, W9 Week 9, W12 Week 12, W24 Weeks 24

**Fig. 4:**
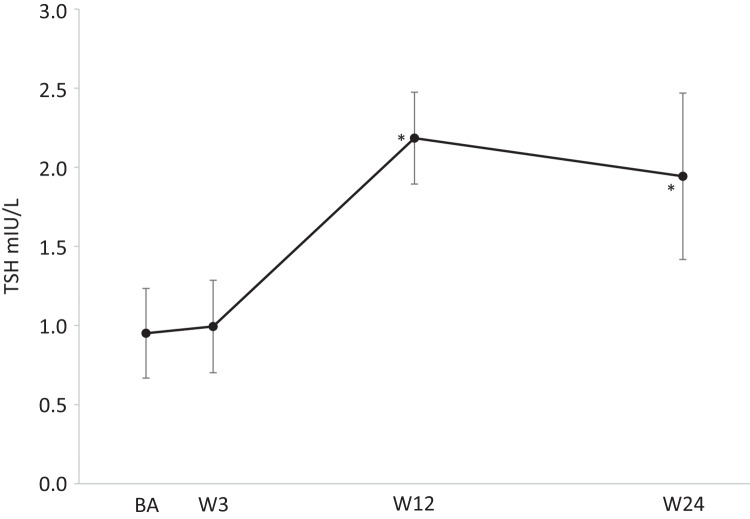
Change in level of TSH throughout the study period. *Statistically significant change from baseline at the 0.05 level. TSH Thyroid stimulating hormone

Regarding thyroid function T3 changed significantly over time (*p* < 0.01) (Fig. 3). At baseline T3 was 1.92 ± 0.67 nmol/L and decreased significantly towards week 3, 12, and 24 to 1.54 ± 0.36 nmol/L, 1.47 ± 0.40 nmol/L, and 1.54 ± 0.26 nmol/L, respectively (*p* < 0.01 for all). Similarly, TSH increased significantly over time (*p* = 0.014) (Fig. 4). At baseline and week 3 TSH was 0.95 ± 0.28 mIU/L and 0.99 ± 0.29 mIU/L, respectively and increased significantly to 2.18 ± 0.29 mIU/L and 1.94 ± 0.53 mIU/L at week 9 and 12, respectively (*p* < 0.05 for both). T4 did not change significantly over the course of the study (*p* = 0.37) (Fig. 3).

### Correlations

We performed correlations between 12- and 24-week changes in TSH, T3 and T4 and 12- and 24-week changes in BMD and BTMs. There were significant and positive correlations between 12-week changes in T3 and T4 and 12-week changes in BTMs (*r* = 0.42–0.52, *p* < 0.05) as well as a significant and negative correlation between 24-week changes in T3 and thBMD (*r* = 0.60, *p* < 0.01). All other correlations, however, were non-significant (Table 4).

**Table 4:** Correlations between 12- and 24 week changes in T3 or T4 and BMD

	ΔlsBMD W12	ΔfnBMD W12	ΔthBMD W12	ΔCTx W12	ΔP1NP W12
ΔT3 W12	−0.12	0.22	−0.28	**0.45**	**0.42**
ΔT4 W12	−0.10	−0.08	−0.23	**0.47**	**0.52**
ΔTSH W12	−0.26	0.00	−0.26	−0.18	0.03

	ΔlsBMD W24	ΔfnBMD W24	ΔthBMD W24	ΔCTx W24	ΔP1NP W24
ΔT3 W24	−0.38	−0.14	−**0.60**	0.40	0.26
ΔT4 W24	−0.01	−0.17	0.00	0.31	0.36
ΔTSH W24	−0.17	0.10	−0.14	−0.01	0.25

Statistically significant correlations are highlighted in boldface

*W12* Week 12, *W24* Week 24, *T3* Tri-iodothyronine, *T4* Thyroxine, *TSH* Thyroid stimulating hormone, *lsBMD* Lumbar spine BMD, *fnBMD* Femoral neck BMD, *thBMD* Total hip BMD

## Discussion

In the present study we performed a comprehensive analysis of the effect of a 12-week course of high dose intravenous MP with concomitant treatment of hyperthyroidism on bone turnover, -mass, and –structure in patients with GO. Our study is largest study on this topic to date and shows that during the 12-week treatment period bone resorption decreased significantly but BMD was unchanged. During 24 weeks of follow up, however, BMD at the hip sites increased significantly. Moreover, cortical porosity decreased significantly and cortical vBMD increased significantly at both the tibia and radius during 12 weeks.

Glucocorticoids affect all types of bone cells. Treatment with GC causes an early and transient activation of osteoclastogenesis owing to an increased production of receptor activator of nuclear factor κB ligand and decreased production of osteoprotegerin by osteoblasts leading to increased resorption [18]. With longer treatment duration osteoblastogenesis decreases [19] and osteoblast apoptosis increases [20] which in turn decreases bone formation. This combined effect of initially increased resorption with loss of BMD followed by decreased formation decreases BMD [9] and increases fracture risk [10]. Thyroid hormones also affect bone. Tri-iodothyronin acts directly on osteoblasts to increase proliferation and differentiation, and osteoclast number and –activity is increased during thyroid hormone excess although this effect may be indirectly mediated by other bone cells [21]. In accordance with this thyrotoxicosis increases bone turnover leading to bone loss [22] and increased fracture risk [23]. A recent study in patients with newly diagnosed Graves´ disease, however, shows that treatment of thyrotoxicosis *increases* BMD at both hip and spine sites [24] and thereby restores bone metabolism. Taken together, in patients like in the present study with Graves’ disease under treatment *and* GO treated with MP there are factors that could affect bone both positively and negatively.

During the course of our study, T3 decreased significantly and TSH increased significantly showing remission of the thyrotoxicosis. Moreover, CTx decreased significantly from baseline and already to 3 weeks, and there was a trend for P1NP to do the same. These findings are in line with a previous study. Thus, in 23 patients with GO following the same treatment with MP as in our study Rymuza et al. found that both CTx and P1NP decreased during 12 weeks [25]. In that study BTMs were monitored more closely during the first week than in our study but with the same decreasing pattern. The course of the thyrotoxicosis during the study, however, was not described. Collectively the studies suggest that either the intermittent high-dose MP has no negative effect on bone mass and -turnover or that this is outweighed by the effect on bone of remission of the thyrotoxicosis. The latter point is corroborated by the positive and significant correlations between 12-week changes in T3 and CTx and P1NP and maybe by the fact that in the stratified analyses the decrease in CTx was only significant in the TSH supressed group.

In line with the decrease in bone turnover, BMD at the hip increased significantly albeit not until 24 weeks. This finding is similar to that in another study in 35 patients with GO treated with MP in which BMD increased beyond least significant change in 43% of patients during a 12-week period [26]. The reason for the seemingly faster increase in BMD could be degree of thyrotoxicosis or the way data was analysed. A smaller Italian study in 11 patients evaluated changes in both BTM and BMD during a 12-month follow up using the same MP treatment regimen and found no change in either parameter. This may be due to less statistical power [27]. In the present study, the improvement in BMD in the analyses stratified for baseline TSH was only statistically significant in the patients with suppressed TSH at baseline. This may be owing to largest power in this group but may also suggest that remission of thyrotoxicosis is important and that the used glucocorticoid regimen probably has limited negative effect on bone health.

Bone structure evaluated by HRpQCT has never been examined in this setting before. Our study showed that cortical porosity *decreased* and cortical vBMD *increased* at both the tibia and radius. Moreover, trabecular area decreased at the tibia.

Longitudinal changes in bone structure as a consequence of GC treatment have been examined in patients with systemic lupus erythematosus treated with daily, oral GC and compared with healthy controls. During 2 years of follow-up cortical porosity increased and cortical area and thickness decreased in the patients. There were no differences in cortical or total vBMD but surprisingly trabecular vBMD decreased more in controls [28]. Bone structure has also been evaluated in patients with thyroid disease. In a study using histomorphomtery, 22 hyperthyroid patients were evaluated before and after 4 months of treatment with antithyroid drugs [29]. During that period, cortical porosity also increased whereas the amount of trabecular bone was unchanged. A recent study evaluated changes in bone structure during treatment of newly diagnosed *hypo*thyroid patients. After attainment of 12 months of euthyroidism cortical porosity had increased and cortical vBMD had decreased at both the tibia and radius. These changes are the exact opposite of what we found in the present study. Moreover, total vBMD at the tibia and trabecular vBMD at the radius decreased [30]. Taken together these studies and our suggest two points. First, intermittent high-dose MP does not impact negatively on bone structure or its effect is outweighed by remission of thyrotoxicosis. Second, bone structure changes during thyroid disease appear to be mostly present in the cortical compartment. The reason for the latter remains to be elucidated but a reason for the finding may be that at least in our study and the study on patients with hypothyroidism, treatment for thyroid disease was initiated before enrolment in the studies. Hence, changes at the more metabolically active trabecular compartment may have occurred prior to the baseline scan.

Strengths of the present study include its comprehensive analysis of bone turnover, density and structure, a sample size allowing for statistical power, and that we treated GO uniformly in all patients. Limitations include that follow up may have been too short to fully elucidate the effect on bone structure since follow up for the HRpQCT scans was only 12 weeks and thereby shorter than a remodelling cycle [31]. Moreover, the design of the study does not directly allow distinction of the effect of MP and treatment of thyrotoxicosis on bone. This fact, however, also makes the study pragmatic and clinically relevant. Finally, we only have 24-week data on a subset of patients.

In conclusion, we investigated the effect of a 12-week course of high dose intravenous MP on bone turnover, -mass, and –structure in patients with GO and found that during the course bone turnover and cortical porosity decreased. Bone mineral density remained stable during 12 weeks but increased at the hip during 24 weeks. The study suggests that the normalisation of thyroid function is a more important determinant for bone health than the high dose MP treatment in patients with GO.
